# Replanting Teeth

**Published:** 1879-05

**Authors:** 


### REPLANTING TEETH.

A correspondent states that in the preparation of the tooth for replanting or transplanting he fills the foramen at the end of the root, by inserting in it a gold screw that will completely fill it, then fills pulp chamber and cavity of decay, then upon the surface of the root cuts a groove from apex to neck, this facilitates the escape of the blood as the tooth is placed in the socket.

This, it is alleged, will obviate the pain that otherwise sometimes accompanies the replacement of a tooth.

This groove would rarely, if ever, be required, in transplanting, from the fact that an absolute adaptation of a root to another socket is never found.

Replanting is becoming more and more a favorite method of treating alveolar abscess.

